# Setting-specific factors influencing trajectories of alcohol consumption in untreated control groups in early intervention studies for problematic drinking

**DOI:** 10.1186/1940-0640-7-S1-A68

**Published:** 2012-10-09

**Authors:** Gallus Bischof, Jennis Freyer-Adam, Christian Meyer, John Ulrich, Hans-Juergen Rumpf

**Affiliations:** 1 Department Of Psychiatry and Psychotherapy, University of Luebeck, Luebeck, Germany; 2 Institute of Epidemiology and Social Medicine, Ernst-Moritz-Arndt-University of Greifswald, Greifswald, Germany

## 

While the efficacy of screening and brief intervention (SBI) for problematic alcohol use has been proven in various outpatient settings, findings from inpatient studies remain inconclusive. Some research has shown that inpatient treatment leads to elevated motivation to change problem behaviors. This study analyzed changes in drinking behavior among untreated patients with problematic alcohol use recruited proactively in general practice (GP) settings and in two general hospitals. We compared problem drinkers randomly assigned to an untreated control group from a study of GP patients (n = 99) with inpatients recruited at the surgical and internal wards of the two general hospitals (n = 173). In both studies, all incoming patients aged 18-64 years were screened for alcohol use. Patients meeting criteria for at-risk drinking according to British Medical Association guidelines (>20-30 g alcohol per day) or meeting criteria for alcohol abuse or dependence according to DSM-IV criteria were included. Both samples received a non-alcohol–specific brochure on healthy living and were re-assessed 12 months later (response rate, 90.4%). The hospitalized patients were older, more often male, had less schooling, and showed elevated readiness to change at baseline compared with GP patients. The groups did not differ concerning alcohol-related diagnoses. At 12-month follow-up, significantly more GH patients than GP patients reported abstinence or low-risk drinking (50.0% versus 26.1%, p < 0.001). Recruitment setting (GH versus GP) remained a significant predictor for nonproblematic drinking or abstinence even after controlling for baseline differences between groups. Findings suggest that natural processes of change from problematic alcohol use are elevated after non-alcohol–related inpatient treatment. This might, in part, explain the lack of findings supporting BI for problematic alcohol use in inpatient settings.

